# Crystal structure of (*Z*)-1-phenyl-3-styryl­undeca-2-en-4,10-diyn-1-ol

**DOI:** 10.1107/S205698901402742X

**Published:** 2015-01-01

**Authors:** Rakesh Ganguly, Sally, Philip Wai Hong Chan

**Affiliations:** a Division of Chemistry & Biological Chemistry, SPMS-CBC-01-18D, Nanyang Technological University, 21 Nanyang Link, 637371, Singapore; b Division of Chemistry & Biological Chemistry, Nanyang Technological University, 21 Nanyang Link, 637371, Singapore

**Keywords:** crystal structure, 1,3-migration, alcohol group, catalytic cyclization, styrylundeca­nene.

## Abstract

The mol­ecule of the title compound, C_25_H_24_O, obtained by acid-catalysed 1,3-migration of an alcohol group, is T-shaped. The planes of the two phenyl rings are inclined to one another by 81.9 (2)°. In the crystal, mol­ecules are linked by O—H⋯O hydrogen bonds, forming chains along [001].

## Related literature   

For the 1,3-migration of an alcoholic group adjacent to a vinyl group in the presence of a Lewis acid, see: Piotti & Alper (1997 ▸); Poloukhtine & Popik (2005 ▸). For catalytic cyclization of alcohols containing a number of unsaturated groups, see: Teo *et al.* (2014 ▸).
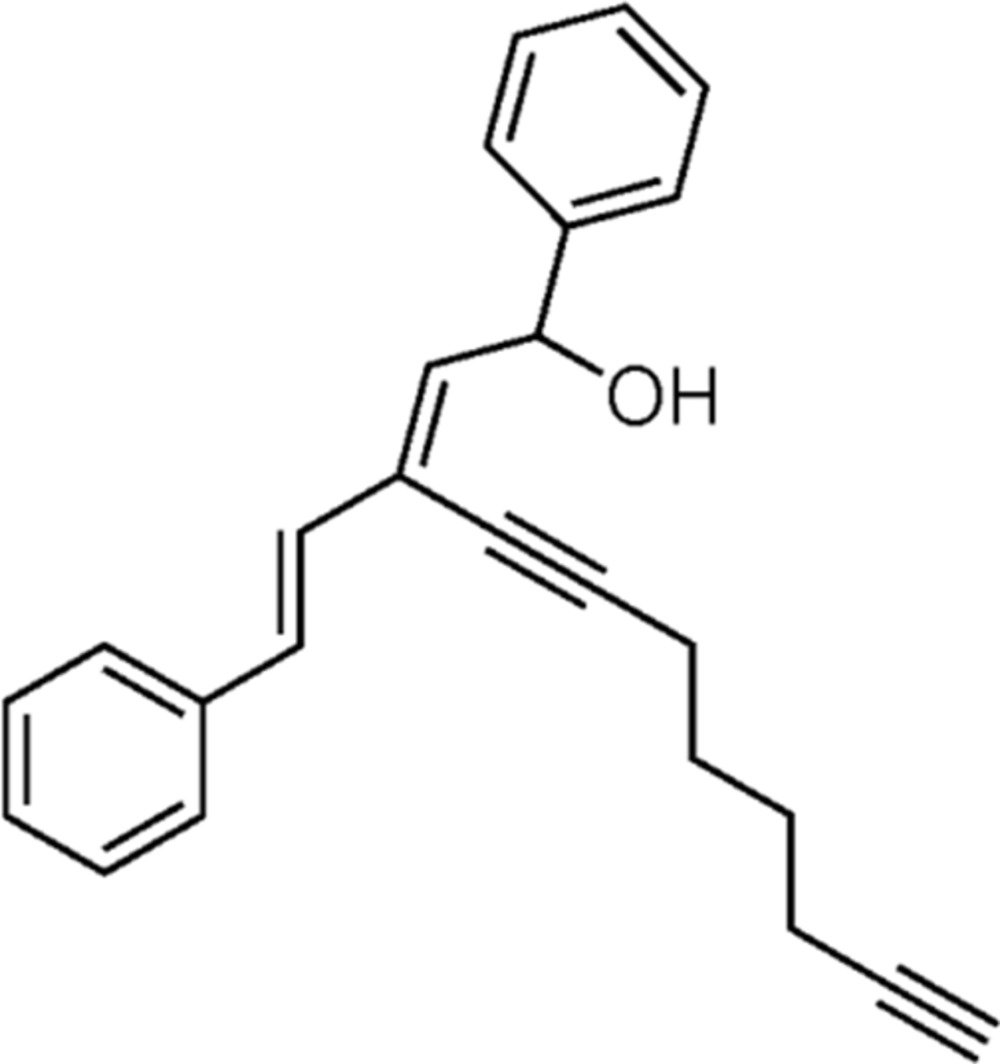


## Experimental   

### Crystal data  

C_25_H_24_O
*M* *_r_* = 340.44
Trigonal, 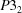

*a* = 17.867 (2) Å
*c* = 5.3290 (6) Å
*V* = 1473.3 (4) Å^3^
*Z* = 3
Mo *K*α radiation
μ = 0.07 mm^−1^
*T* = 103 K
0.34 × 0.04 × 0.04 mm

### Data collection  

Bruker Kappa APEXII CCD diffractometer
Absorption correction: multi-scan (*SADABS*; Bruker, 2013 ▸) *T*_min_ = 0.74, *T*_max_ = 1.00
14182 measured reflections
4846 independent reflections
2945 reflections with *I* > 2σ(*I*)
*R*_int_ = 0.078

### Refinement  

*R*[*F* ^2^ > 2σ(*F* ^2^)] = 0.058
*wR*(*F* ^2^) = 0.129
*S* = 0.98
4846 reflections
235 parameters
1 restraint
H-atom parameters constrained
Δρ_max_ = 0.39 e Å^−3^
Δρ_min_ = −0.24 e Å^−3^

### 

Data collection: *APEX2* (Bruker, 2013 ▸); cell refinement: *SAINT* (Bruker, 2013 ▸); data reduction: *SAINT*; program(s) used to solve structure: *SHELXS97* (Sheldrick 2008 ▸); program(s) used to refine structure: *SHELXL2014* (Sheldrick, 2008 ▸); molecular graphics: *ORTEP-3 for Windows* (Farrugia, 2012 ▸) and *Mercury* (Macrae *et al.*, 2008 ▸); software used to prepare material for publication: *SHELXL2014*, *PLATON* (Spek, 2009 ▸) and *publCIF* (Westrip, 2010 ▸).

## Supplementary Material

Crystal structure: contains datablock(s) I, New_Global_Publ_Block. DOI: 10.1107/S205698901402742X/su5038sup1.cif

Structure factors: contains datablock(s) I. DOI: 10.1107/S205698901402742X/su5038Isup2.hkl

Supporting information file. DOI: 10.1107/S205698901402742X/su5038Isup3.cml

**Fig. 1.**
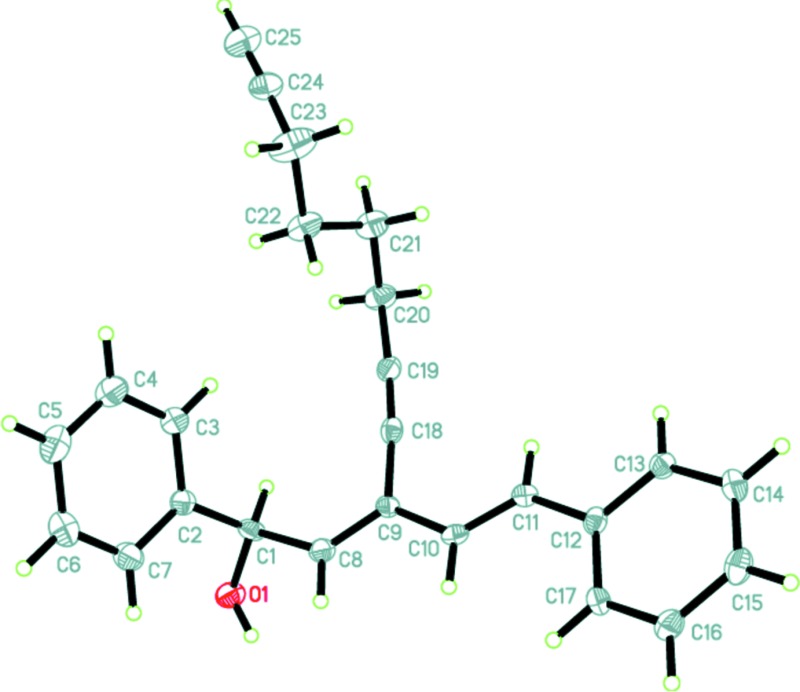
A view of the molecular structure of the title compound, with atom labelling. Displacement ellipsoids are drawn at the 50% probability level.

**Fig. 2.**
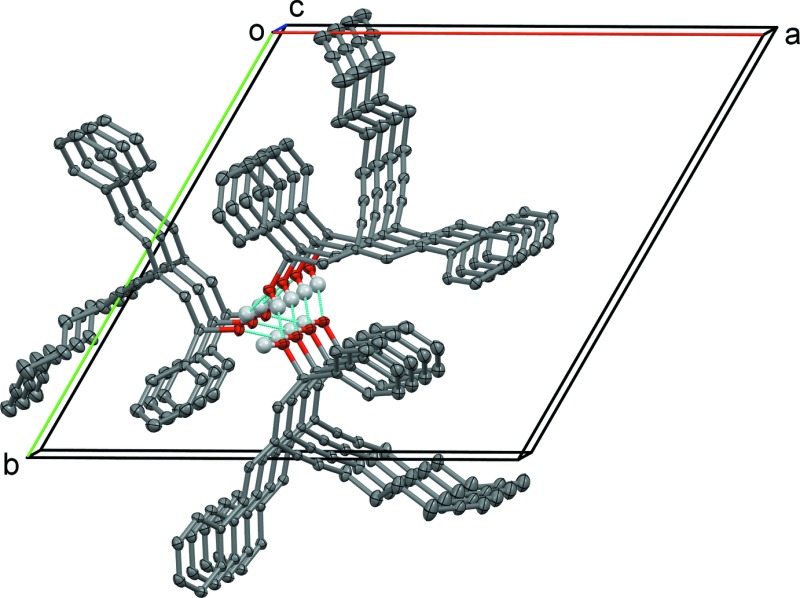
A partial view along the c axis of the crystal packing of the title compound. Hydrogen bonds are shown as dashed lines (see Table 1 for details).

. DOI: 10.1107/S205698901402742X/su5038fig1.tif
A view of the mol­ecular structure of the title compound, with atom labelling. Displacement ellipsoids are drawn at the 50% probability level.

c . DOI: 10.1107/S205698901402742X/su5038fig2.tif
A partial view along the *c* axis of the crystal packing of the title compound. Hydrogen bonds are shown as dashed lines (see Table 1 for details).

**Fig. 3.**
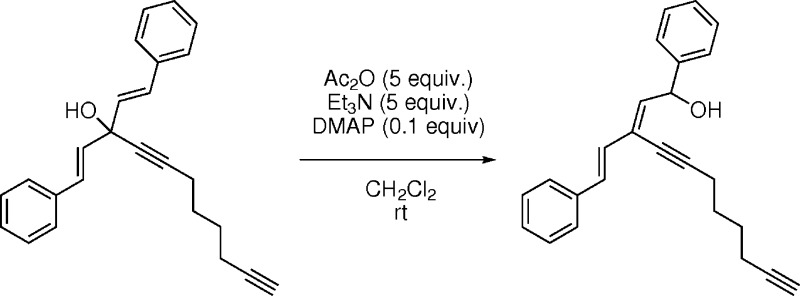
Reaction scheme.

. DOI: 10.1107/S205698901402742X/su5038fig3.tif
Reaction scheme.

CCDC reference: 1009363

Additional supporting information:  crystallographic information; 3D view; checkCIF report

## Figures and Tables

**Table 1:** Hydrogen-bond geometry (, )

*D*H*A*	*D*H	H*A*	*D* *A*	*D*H*A*
O1H1*A*O1^i^	0.84	1.83	2.652(3)	166

Symmetry code: (i) 
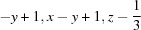
.

## supplementary crystallographic information

### S1. Comment

1,3-migration of an alcoholic group adjacent to a vinyl group in the presence of a Lewis-acid is widely known (Piotti & Alper, 1997). One such example was demonstrated recently in the preparation of 4-(α-hy­droxy­benzyl)-1-*tert*-butyl­dimethyl­silyl­oxy-4-cyclo­decene-2,6-diyne (Poloukhtine & Popik, 2005). In addition, alcohols containing many unsaturated groups provide an access to a myriad of types of functionalization such as catalytic cyclization (Teo *et al.*, 2014). Herein, we report on the synthesis and crystal structure of the title compound, obtained by the acid-catalyzed 1,3-migration of an alcoholic group.

The molecular structure of the title compound is illustrated in Fig. 1. The molecule is T-shaped with the two phenyl ring inclined to one another by 81.9 (2) °.

In the crystal, molecules are linked by O—H···O hydrogen bonds forming chains along the *c* axis direction (Table 1 and Fig. 2).

### S2. Synthesis and crystallization

The synthesis of the title compound is illustrated in Fig. 3. (*Z*)-1-phenyl-3-styrylundeca-1-en-4,10-diyn-3-ol (1mmol, 340.5 mg) was dissolved in 10 ml of CH_2_Cl_2_. DMAP [4-(di­methyl­amino)­pyridine; 0.1 mmol, 12 mg], tri­ethyl­amine (5 mmol, 0.70 ml) and acetic anhydride (5 mmol, 0.47 ml) were added sequentially and the reaction mixture was stirred overnight. It was then washed with saturated sodium bicarbonate and extracted twice with CH_2_Cl_2_. The organic layers were combined, dried with MgSO_4_ and the solvent was removed under reduced pressure. The resulting oil was purified by column chromatography with hexane/ethyl­acetate as eluent. The product was recrystallized with ethyl­acetate to give a colourless compound in 80% yield. Slow evaporation of a solution in ethyl­acetate gave needle-like crystals. ^1^H NMR (400 MHz, CDCl_3_) 1.71–1.83 (m, 4H), 1.97 (t, 1H), 2.20 (s, 1H), 2.26–2.30 (m, 2H), 2.55 (t, 2), 5.90 (d, 1H), 6.09 (d, 1H), 6.68 (d, 1H), 6.99 (d, 1H), 7.21–7.47 (m, 10H) 13 C NMR (100 MHz, CDCl3) 18.0, 19.2, 27.7, 27.7, 68.8, 72.5, 75.3, 84.0, 97.9, 124.1, 125.9, 126.8, 127.7, 127.9, 128.6, 128.6, 132.4, 136.8, 140.4, 142.7

### S3. Refinement

The OH and C-bound H atoms were included in calculated positions and treated as riding atoms: O—H = 0.84 Å, C—H = 0.95 - 1.00 Å with U_iso_(H) = 1.5U_eq_(O) for the OH H atom and = 1.2U_eq_(C) for other H atoms.

### Crystal data

**Table:**

C_25_H_24_O	*D*_x_ = 1.151 Mg m^−^^3^
*M**_r_* = 340.44	Mo *K*α radiation, λ = 0.71073 Å
Trigonal, *P*3_2_	Cell parameters from 1285 reflections
*a* = 17.867 (2) Å	θ = 2.3–20.5°
*c* = 5.3290 (6) Å	µ = 0.07 mm^−^^1^
*V* = 1473.3 (4) Å^3^	*T* = 103 K
*Z* = 3	Needle, colourless
*F*(000) = 546	0.34 × 0.04 × 0.04 mm

### Data collection

**Table:**

Bruker Kappa APEXII CCD diffractometer	4846 independent reflections
Radiation source: fine-focus sealed tube	2945 reflections with *I* > 2σ(*I*)
Graphite monochromator	*R*_int_ = 0.078
ω and φ scan	θ_max_ = 28.3°, θ_min_ = 2.6°
Absorption correction: multi-scan (*SADABS*; Bruker, 2013)	*h* = −23→14
*T*_min_ = 0.74, *T*_max_ = 1.00	*k* = −22→23
14182 measured reflections	*l* = −7→7

### Refinement

**Table:**

Refinement on *F*^2^	Primary atom site location: structure-invariant direct methods
Least-squares matrix: full	Secondary atom site location: difference Fourier map
*R*[*F*^2^ > 2σ(*F*^2^)] = 0.058	Hydrogen site location: inferred from neighbouring sites
*wR*(*F*^2^) = 0.129	H-atom parameters constrained
*S* = 0.98	*w* = 1/[σ^2^(*F*_o_^2^) + (0.0486*P*)^2^] where *P* = (*F*_o_^2^ + 2*F*_c_^2^)/3
4846 reflections	(Δ/σ)_max_ < 0.001
235 parameters	Δρ_max_ = 0.39 e Å^−^^3^
1 restraint	Δρ_min_ = −0.24 e Å^−^^3^

### Special details

**Table:**

Geometry. All e.s.d.'s (except the e.s.d. in the dihedral angle between two l.s. planes) are estimated using the full covariance matrix. The cell e.s.d.'s are taken into account individually in the estimation of e.s.d.'s in distances, angles and torsion angles; correlations between e.s.d.'s in cell parameters are only used when they are defined by crystal symmetry. An approximate (isotropic) treatment of cell e.s.d.'s is used for estimating e.s.d.'s involving l.s. planes.

### Fractional atomic coordinates and isotropic or equivalent isotropic displacement parameters (Å^2^)

**Table:**

	*x*	*y*	*z*	*U*_iso_*/*U*_eq_	
O1	0.30859 (17)	0.59437 (17)	0.3483 (5)	0.0251 (7)	
H1A	0.3463	0.6311	0.2521	0.038*	
C1	0.3069 (3)	0.5136 (2)	0.3226 (7)	0.0199 (9)	
H1	0.3048	0.4898	0.4940	0.024*	
C2	0.2281 (2)	0.4484 (3)	0.1774 (7)	0.0197 (9)	
C3	0.1897 (3)	0.3614 (3)	0.2407 (8)	0.0281 (10)	
H3	0.2105	0.3441	0.3801	0.034*	
C4	0.1209 (3)	0.2999 (3)	0.1007 (9)	0.0341 (12)	
H4	0.0948	0.2407	0.1461	0.041*	
C5	0.0899 (3)	0.3235 (3)	−0.1032 (9)	0.0333 (11)	
H5	0.0434	0.2809	−0.1998	0.040*	
C6	0.1275 (3)	0.4099 (3)	−0.1651 (8)	0.0305 (11)	
H6	0.1060	0.4269	−0.3038	0.037*	
C7	0.1962 (3)	0.4721 (3)	−0.0270 (8)	0.0272 (10)	
H7	0.2216	0.5313	−0.0722	0.033*	
C8	0.3878 (2)	0.5282 (2)	0.1948 (7)	0.0196 (9)	
H8	0.4050	0.5639	0.0497	0.024*	
C9	0.4386 (2)	0.4964 (2)	0.2634 (7)	0.0160 (8)	
C10	0.5143 (2)	0.5153 (2)	0.1121 (7)	0.0171 (8)	
H10	0.5253	0.5510	−0.0311	0.021*	
C11	0.5691 (2)	0.4869 (2)	0.1575 (7)	0.0171 (8)	
H11	0.5608	0.4552	0.3082	0.021*	
C12	0.6413 (2)	0.5000 (2)	−0.0039 (7)	0.0173 (9)	
C13	0.6850 (2)	0.4550 (2)	0.0458 (7)	0.0184 (9)	
H13	0.6692	0.4182	0.1882	0.022*	
C14	0.7511 (3)	0.4630 (3)	−0.1091 (8)	0.0220 (9)	
H14	0.7805	0.4324	−0.0718	0.026*	
C15	0.7739 (3)	0.5160 (3)	−0.3182 (7)	0.0245 (10)	
H15	0.8184	0.5209	−0.4264	0.029*	
C16	0.7321 (2)	0.5620 (3)	−0.3704 (7)	0.0231 (9)	
H16	0.7482	0.5985	−0.5134	0.028*	
C17	0.6665 (3)	0.5545 (3)	−0.2137 (7)	0.0204 (9)	
H17	0.6385	0.5867	−0.2492	0.024*	
C18	0.4152 (2)	0.4385 (3)	0.4758 (8)	0.0184 (8)	
C19	0.3892 (2)	0.3880 (2)	0.6457 (7)	0.0201 (9)	
C20	0.3519 (3)	0.3267 (3)	0.8553 (8)	0.0275 (10)	
H20A	0.3052	0.3336	0.9313	0.033*	
H20B	0.3973	0.3426	0.9844	0.033*	
C21	0.3157 (3)	0.2328 (3)	0.7913 (8)	0.0310 (11)	
H21A	0.2888	0.1977	0.9434	0.037*	
H21B	0.3637	0.2235	0.7405	0.037*	
C22	0.2484 (3)	0.2009 (3)	0.5807 (8)	0.0317 (11)	
H22A	0.2021	0.2137	0.6248	0.038*	
H22B	0.2760	0.2322	0.4237	0.038*	
C23	0.2091 (3)	0.1044 (3)	0.5383 (9)	0.0467 (14)	
H23A	0.1740	0.0881	0.3830	0.056*	
H23B	0.2562	0.0913	0.5127	0.056*	
C24	0.1549 (3)	0.0521 (3)	0.7450 (9)	0.0326 (11)	
C25	0.1097 (3)	0.0105 (3)	0.9069 (10)	0.0431 (13)	
H25	0.0727	−0.0235	1.0394	0.052*	

### Atomic displacement parameters (Å^2^)

**Table:**

	*U*^11^	*U*^22^	*U*^33^	*U*^12^	*U*^13^	*U*^23^
O1	0.0252 (16)	0.0188 (15)	0.0341 (17)	0.0130 (13)	0.0087 (14)	0.0019 (13)
C1	0.024 (2)	0.021 (2)	0.021 (2)	0.0159 (19)	0.0076 (18)	0.0067 (18)
C2	0.019 (2)	0.021 (2)	0.024 (2)	0.0129 (18)	0.0084 (18)	0.0036 (18)
C3	0.022 (2)	0.025 (2)	0.036 (3)	0.010 (2)	0.002 (2)	0.008 (2)
C4	0.026 (2)	0.026 (3)	0.047 (3)	0.010 (2)	0.003 (2)	0.005 (2)
C5	0.024 (2)	0.034 (3)	0.038 (3)	0.011 (2)	−0.001 (2)	−0.006 (2)
C6	0.031 (3)	0.035 (3)	0.030 (3)	0.020 (2)	−0.005 (2)	−0.002 (2)
C7	0.028 (2)	0.027 (2)	0.029 (2)	0.016 (2)	0.003 (2)	0.005 (2)
C8	0.020 (2)	0.018 (2)	0.021 (2)	0.0093 (18)	0.0026 (17)	0.0022 (17)
C9	0.016 (2)	0.0124 (19)	0.019 (2)	0.0071 (17)	−0.0012 (17)	−0.0009 (16)
C10	0.018 (2)	0.0110 (19)	0.019 (2)	0.0051 (17)	0.0007 (17)	0.0011 (16)
C11	0.019 (2)	0.015 (2)	0.017 (2)	0.0078 (18)	0.0010 (17)	0.0004 (16)
C12	0.014 (2)	0.017 (2)	0.020 (2)	0.0066 (17)	−0.0008 (17)	−0.0034 (17)
C13	0.018 (2)	0.019 (2)	0.018 (2)	0.0087 (18)	0.0014 (17)	0.0001 (17)
C14	0.021 (2)	0.025 (2)	0.024 (2)	0.0145 (19)	−0.0020 (18)	−0.0037 (19)
C15	0.019 (2)	0.031 (2)	0.023 (2)	0.012 (2)	0.0035 (18)	−0.0035 (19)
C16	0.021 (2)	0.025 (2)	0.022 (2)	0.0096 (19)	0.0035 (18)	0.0020 (19)
C17	0.017 (2)	0.021 (2)	0.025 (2)	0.0119 (18)	−0.0006 (18)	0.0014 (18)
C18	0.015 (2)	0.020 (2)	0.021 (2)	0.0094 (17)	−0.0002 (17)	−0.0042 (18)
C19	0.017 (2)	0.020 (2)	0.021 (2)	0.0073 (18)	0.0002 (18)	0.0011 (18)
C20	0.031 (3)	0.024 (2)	0.020 (2)	0.008 (2)	−0.001 (2)	0.0045 (19)
C21	0.033 (3)	0.026 (2)	0.027 (2)	0.010 (2)	0.002 (2)	0.002 (2)
C22	0.032 (3)	0.024 (2)	0.029 (3)	0.006 (2)	0.003 (2)	0.003 (2)
C23	0.055 (3)	0.028 (3)	0.039 (3)	0.007 (2)	0.006 (3)	−0.002 (2)
C24	0.033 (3)	0.019 (2)	0.043 (3)	0.010 (2)	−0.004 (2)	−0.003 (2)
C25	0.037 (3)	0.029 (3)	0.054 (4)	0.009 (2)	0.001 (3)	0.006 (3)

### Geometric parameters (Å, º)

**Table:**

O1—C1	1.435 (4)	C13—C14	1.388 (5)
O1—H1A	0.8400	C13—H13	0.9500
C1—C8	1.498 (5)	C14—C15	1.385 (6)
C1—C2	1.516 (6)	C14—H14	0.9500
C1—H1	1.0000	C15—C16	1.388 (6)
C2—C3	1.390 (5)	C15—H15	0.9500
C2—C7	1.390 (5)	C16—C17	1.390 (5)
C3—C4	1.387 (6)	C16—H16	0.9500
C3—H3	0.9500	C17—H17	0.9500
C4—C5	1.378 (6)	C18—C19	1.197 (5)
C4—H4	0.9500	C19—C20	1.470 (5)
C5—C6	1.381 (6)	C20—C21	1.504 (6)
C5—H5	0.9500	C20—H20A	0.9900
C6—C7	1.385 (6)	C20—H20B	0.9900
C6—H6	0.9500	C21—C22	1.531 (6)
C7—H7	0.9500	C21—H21A	0.9900
C8—C9	1.341 (5)	C21—H21B	0.9900
C8—H8	0.9500	C22—C23	1.519 (6)
C9—C18	1.446 (5)	C22—H22A	0.9900
C9—C10	1.461 (5)	C22—H22B	0.9900
C10—C11	1.332 (5)	C23—C24	1.456 (7)
C10—H10	0.9500	C23—H23A	0.9900
C11—C12	1.467 (5)	C23—H23B	0.9900
C11—H11	0.9500	C24—C25	1.161 (6)
C12—C13	1.399 (5)	C25—H25	0.9500
C12—C17	1.401 (5)		
			
C1—O1—H1A	109.5	C12—C13—H13	119.4
O1—C1—C8	109.5 (3)	C15—C14—C13	119.5 (4)
O1—C1—C2	111.5 (3)	C15—C14—H14	120.2
C8—C1—C2	110.3 (3)	C13—C14—H14	120.2
O1—C1—H1	108.5	C14—C15—C16	120.3 (4)
C8—C1—H1	108.5	C14—C15—H15	119.9
C2—C1—H1	108.5	C16—C15—H15	119.9
C3—C2—C7	118.8 (4)	C15—C16—C17	120.1 (4)
C3—C2—C1	119.0 (4)	C15—C16—H16	120.0
C7—C2—C1	122.0 (4)	C17—C16—H16	120.0
C2—C3—C4	120.2 (4)	C16—C17—C12	120.6 (4)
C2—C3—H3	119.9	C16—C17—H17	119.7
C4—C3—H3	119.9	C12—C17—H17	119.7
C5—C4—C3	120.9 (4)	C19—C18—C9	174.8 (4)
C5—C4—H4	119.5	C18—C19—C20	176.0 (4)
C3—C4—H4	119.5	C19—C20—C21	116.2 (4)
C4—C5—C6	119.0 (4)	C19—C20—H20A	108.2
C4—C5—H5	120.5	C21—C20—H20A	108.2
C6—C5—H5	120.5	C19—C20—H20B	108.2
C5—C6—C7	120.7 (4)	C21—C20—H20B	108.2
C5—C6—H6	119.6	H20A—C20—H20B	107.4
C7—C6—H6	119.6	C20—C21—C22	113.6 (4)
C6—C7—C2	120.4 (4)	C20—C21—H21A	108.8
C6—C7—H7	119.8	C22—C21—H21A	108.8
C2—C7—H7	119.8	C20—C21—H21B	108.8
C9—C8—C1	126.9 (3)	C22—C21—H21B	108.8
C9—C8—H8	116.5	H21A—C21—H21B	107.7
C1—C8—H8	116.5	C23—C22—C21	111.3 (4)
C8—C9—C18	120.1 (3)	C23—C22—H22A	109.4
C8—C9—C10	119.7 (3)	C21—C22—H22A	109.4
C18—C9—C10	120.0 (3)	C23—C22—H22B	109.4
C11—C10—C9	125.7 (4)	C21—C22—H22B	109.4
C11—C10—H10	117.1	H22A—C22—H22B	108.0
C9—C10—H10	117.1	C24—C23—C22	113.4 (4)
C10—C11—C12	125.9 (4)	C24—C23—H23A	108.9
C10—C11—H11	117.0	C22—C23—H23A	108.9
C12—C11—H11	117.0	C24—C23—H23B	108.9
C13—C12—C17	118.2 (3)	C22—C23—H23B	108.9
C13—C12—C11	119.7 (4)	H23A—C23—H23B	107.7
C17—C12—C11	122.1 (3)	C25—C24—C23	178.0 (5)
C14—C13—C12	121.3 (4)	C24—C25—H25	180.0
C14—C13—H13	119.4		
			
O1—C1—C2—C3	−145.9 (3)	C8—C9—C10—C11	−179.1 (4)
C8—C1—C2—C3	92.2 (4)	C18—C9—C10—C11	−3.5 (6)
O1—C1—C2—C7	38.1 (5)	C9—C10—C11—C12	175.0 (3)
C8—C1—C2—C7	−83.8 (4)	C10—C11—C12—C13	−169.7 (4)
C7—C2—C3—C4	0.2 (6)	C10—C11—C12—C17	8.1 (6)
C1—C2—C3—C4	−175.9 (4)	C17—C12—C13—C14	−0.6 (5)
C2—C3—C4—C5	0.4 (6)	C11—C12—C13—C14	177.2 (3)
C3—C4—C5—C6	−1.0 (7)	C12—C13—C14—C15	−0.6 (6)
C4—C5—C6—C7	1.0 (7)	C13—C14—C15—C16	1.1 (6)
C5—C6—C7—C2	−0.3 (7)	C14—C15—C16—C17	−0.5 (6)
C3—C2—C7—C6	−0.2 (6)	C15—C16—C17—C12	−0.8 (6)
C1—C2—C7—C6	175.8 (4)	C13—C12—C17—C16	1.3 (5)
O1—C1—C8—C9	133.8 (4)	C11—C12—C17—C16	−176.5 (4)
C2—C1—C8—C9	−103.1 (4)	C19—C20—C21—C22	−54.5 (5)
C1—C8—C9—C18	2.6 (6)	C20—C21—C22—C23	−175.6 (4)
C1—C8—C9—C10	178.2 (3)	C21—C22—C23—C24	68.5 (6)

### Hydrogen-bond geometry (Å, º)

**Table:**

*D*—H···*A*	*D*—H	H···*A*	*D*···*A*	*D*—H···*A*
O1—H1*A*···O1^i^	0.84	1.83	2.652 (3)	166

Symmetry code: (i) −*y*+1, *x*−*y*+1, *z*−1/3.
