# Supplementary figures and images for: Crystal structure of (Z)-1-phenyl-3-styryl­undeca-2-en-4,10-diyn-1-ol
Source: Acta Crystallogr E Crystallogr Commun. 2015 Jan 1;71(Pt 1):o64. doi: 10.1107/S205698901402742X (PMC4331854; doi:10.1107/S205698901402742X)

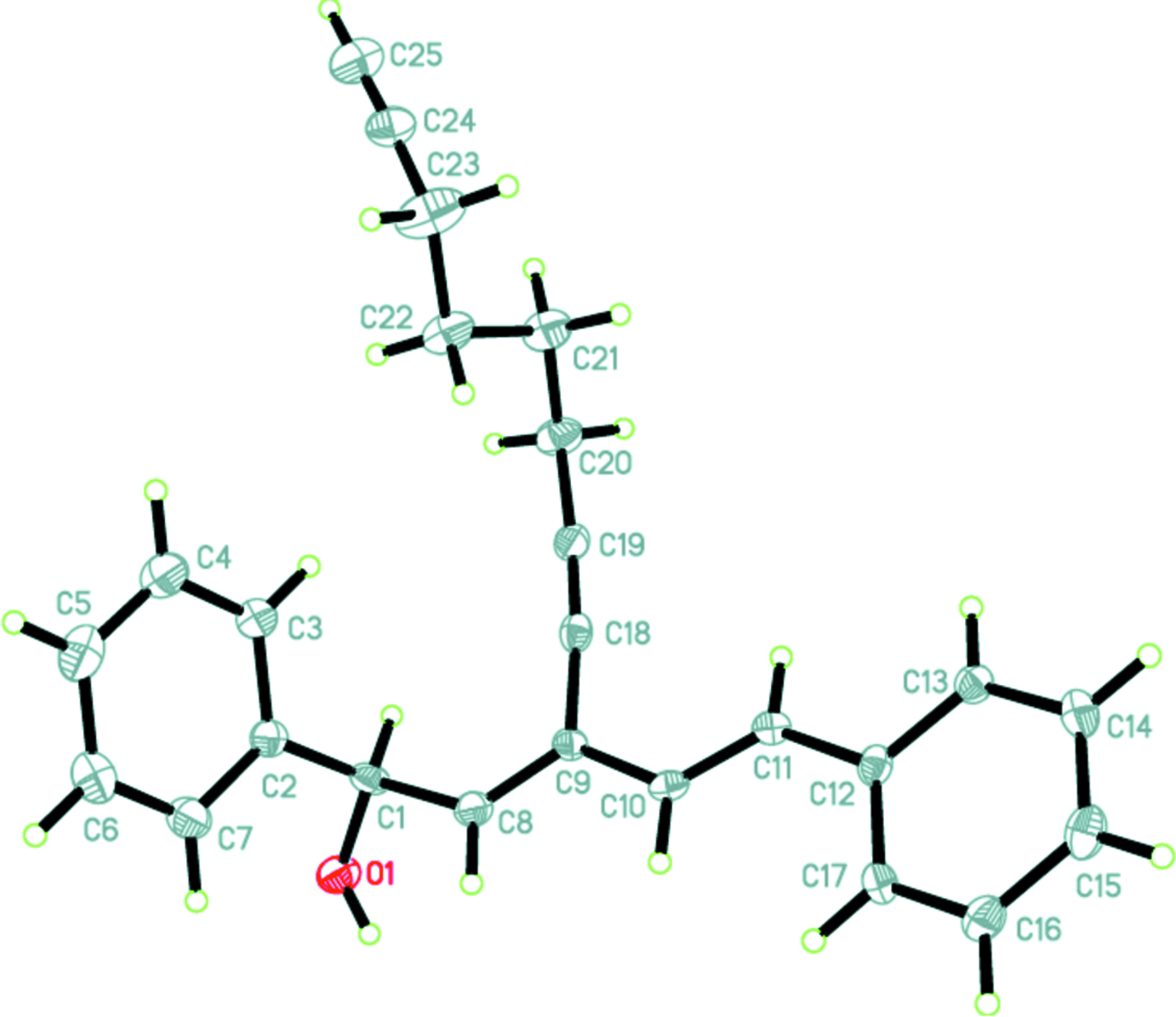

Supplement: Supplementary file 4 [file e-71-00o64-fig1.tif]

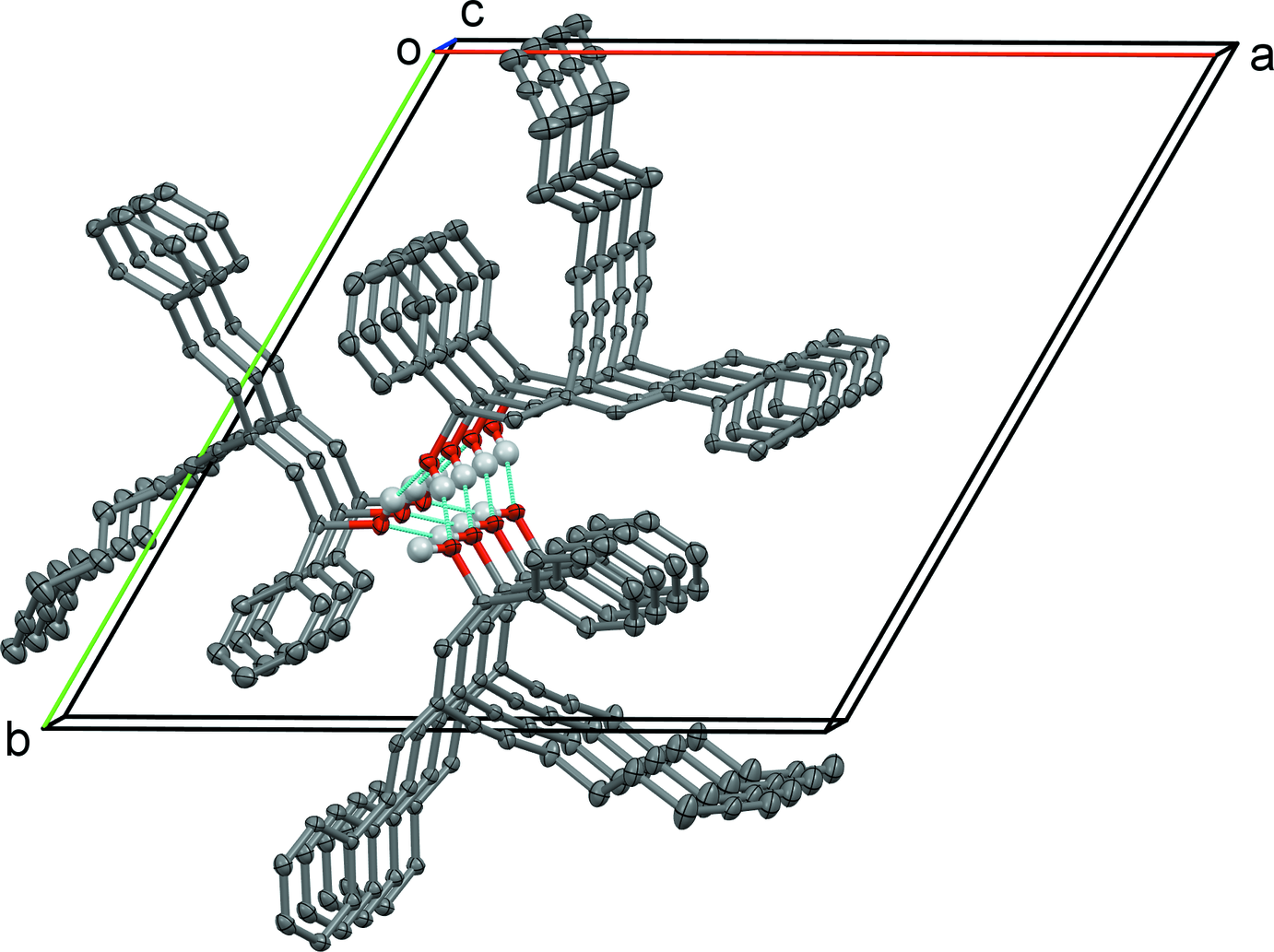

Supplement: Supplementary file 5 [file e-71-00o64-fig2.tif]
